# New hemostatic device for grade IV–V liver injury in porcine model: a proof of concept

**DOI:** 10.1186/s13017-019-0277-7

**Published:** 2019-12-16

**Authors:** Juan José Segura-Sampedro, Cristina Pineño-Flores, Andrea Craus-Miguel, Rafael Morales-Soriano, Francesc Xavier González-Argente

**Affiliations:** 1 General & Digestive Surgery Department, University Hospital Son Espases, Palma de Mallorca, Spain; 2 Health Research Institute of Balearic Islands, Palma de Mallorca, Spain; 3 School of Medicine, University of Balearic Islands, Palma de Mallorca, Spain

**Keywords:** Abdominal trauma, Liver injury, Hemostatic device, Damage control

## Abstract

**Background:**

The liver is the most injured organ following abdominal trauma. Uncontrolled bleeding remains the main cause of early liver injury-related death, with a mortality rate of 50–54% in the first 24 h after admission and with 80% of operative deaths. Packing and reoperation account for the increased survival in severe liver trauma, and they are recommended for severe liver injuries (grades IV–V).

Perihepatic packing can lead to several potential complications. An excessive packing can cause complications due to abdominal compartment syndrome, while a soft packing may be ineffective, and thus, bleeding can continue inadvertently with the consequent hypovolemic shock and potentially death.

**Methods:**

We designed a new vacuum-based device to perform perihepatic packing without the negative side-effects of the classic technique. We conducted a prospective pilot feasibility study in a porcine model. We compared the traditional perihepatic packing (PHP) (*n* = 2) with the new VacBagPack device (VBP) (*n* = 2).

**Results:**

Both pigs survived with the new device and showed an equivalent outcome to the one that survived in the traditional technique group. Blood tests were similar too. This suggests that VBP could be at least as effective as traditional PHP.

**Conclusions:**

We establish a first step towards the development of a new packing device. A new study with a bigger sample size still in pigs will be conducted. Also, an industrial model of the device is currently in production.

## Introduction

The liver is the most injured organ following abdominal trauma. A major liver injury is the leading cause of death in patients with abdominal trauma, the treatment of which still poses a challenge to surgeons [1–3]. Uncontrolled bleeding remains the main cause of early liver injury-related death, with a mortality rate of 50–54% in the first 24 h after admission and with 80% of operative deaths [1, 4, 5].

Packing and reoperation account for the increased survival in severe liver trauma in the last decades [2, 6]. This once revolutionary approach stands now as part of the recommended management for severe liver injuries (grades IV–V) [2, 7].

Although hepatic packing is an extended maneuver, it requires experience to perform it properly and involves several potential complications. An excessive packing can cause complications due to abdominal compartment syndrome [8], while a soft packing may be ineffective and thus bleeding can continue inadvertently with the consequent hypovolemic shock and potentially death [2, 7, 9].

Even if bleeding control is achieved through this maneuver, subsequent complications such as intra-abdominal abscesses, bleeding after withdrawal, or the loss of compresses within the patient are still common.

The purpose of this work is to test a new device conceived to achieve effective bleeding control after severe liver injury without the previous complications. To do so, we compared the classical packing method with the new device in a porcine model.

## Material and methods

We conducted a pilot feasibility study in a porcine model. We compared the traditional perihepatic packing (PHP) (*n* = 2) with the new VacBagPack device (VBP) (*n* = 2).

### VacBagPack device design

This new device consists of a new bag for organ packing that allows compressing the liver in a controlled manner. To that end, the bag is configured to be able to use an external vacuum source to compress the liver via the application of a vacuum, and thus to promote its recovery after a traumatic incident. Furthermore, it may allow determining the volume of blood loss of an organ by connecting a collector to the external vacuum source, which helps determine blood loss at all times [10].

The bag, which has a similar shape to a human liver, may surround the injured portion of the liver. The shape of the bag can be adapted to the wrapped portion of the organ by applying negative pressure, as this allows the bag to compress and thus to adopt the shape of the portion of the organ [10].

The bag is made from a multilayer material that comprises the following elements: an inner layer, an outer layer, and an intermediate layer arranged between the inner and the outer layers [10]. Below, each of these layers is defined in more detail:

- Inner layer
  This is a microperforated inner layer to allow applying the vacuum to the liver [10].
- Outer layer
  This is a watertight outer layer which surrounds the microperforated inner layer. The watertight outer layer may be flexible and comprise an inlet to apply the vacuum to the intermediate region between the microperforated inner layer and the watertight outer layer. Alternatively, the watertight outer layer may lack a preformed inlet, so that the surgeon may perforate, for example with a scalpel or scissors, the outer layer in the most appropriate region to connect the external vacuum source. In this case, the outer layer may be connected to the external vacuum source using known systems, as a VAC Therapy System from KCI Medical [10].
- Intermediate layer
  This is a porous intermediate layer that fills the intermediate region between the microperforated inner layer and the watertight outer layer. The pores of this layer are precisely interconnected so a fluid can easily be distributed throughout the intermediate layer.
  The main objective of this porous intermediate layer is to allow the distribution of the vacuum applied through the outer layer, for example, through its inlet, throughout the entire surface of the inner layer. Because this layer is made from a porous material, it contributes to create a gap between the inner and the outer layers that allows the vacuum to reach all the holes of the inner layer. Furthermore, this configuration also allows the blood extracted from the organ to flow towards the external layer. In addition, the air extracted from the porous intermediate layer along with the effect of the compression of this intermediate layer between the other two layers increase the consistency of the porous layer, which helps hold and compress the liver. Also, the intermediate layer may be made from a compressible and moldable material. The compression of the intermediate layer when a negative pressure is applied uniformly distributes the pressure exerted onto the organ. Moreover, this compressibility also helps control the pressure applied to the organ [10].

When the bag surrounds the liver, this configuration allows the application of vacuum through the watertight outer layer to the intermediate region in order to compress the organ and to extract fluids placed on the surface of the liver. Accordingly, the pressure exerted against the organ may be controlled.

A homemade device was created using compresses, drains, and adhesive plastics as can be seen in the Additional file 1: Video 1.

### Animals

Animal use and procedures were approved by the Research Ethics Committee of the Balearic Islands. The swine model is widely preferred to emulate human trauma [11, 12]. The study was performed in compliance with the Helsinki convention for the use and care of animals. In this study, we used 4 healthy male pigs, aged 3–6 months, which were fasted for 72 h at the start of the investigation.

### Procedure

**Fig. 1:**
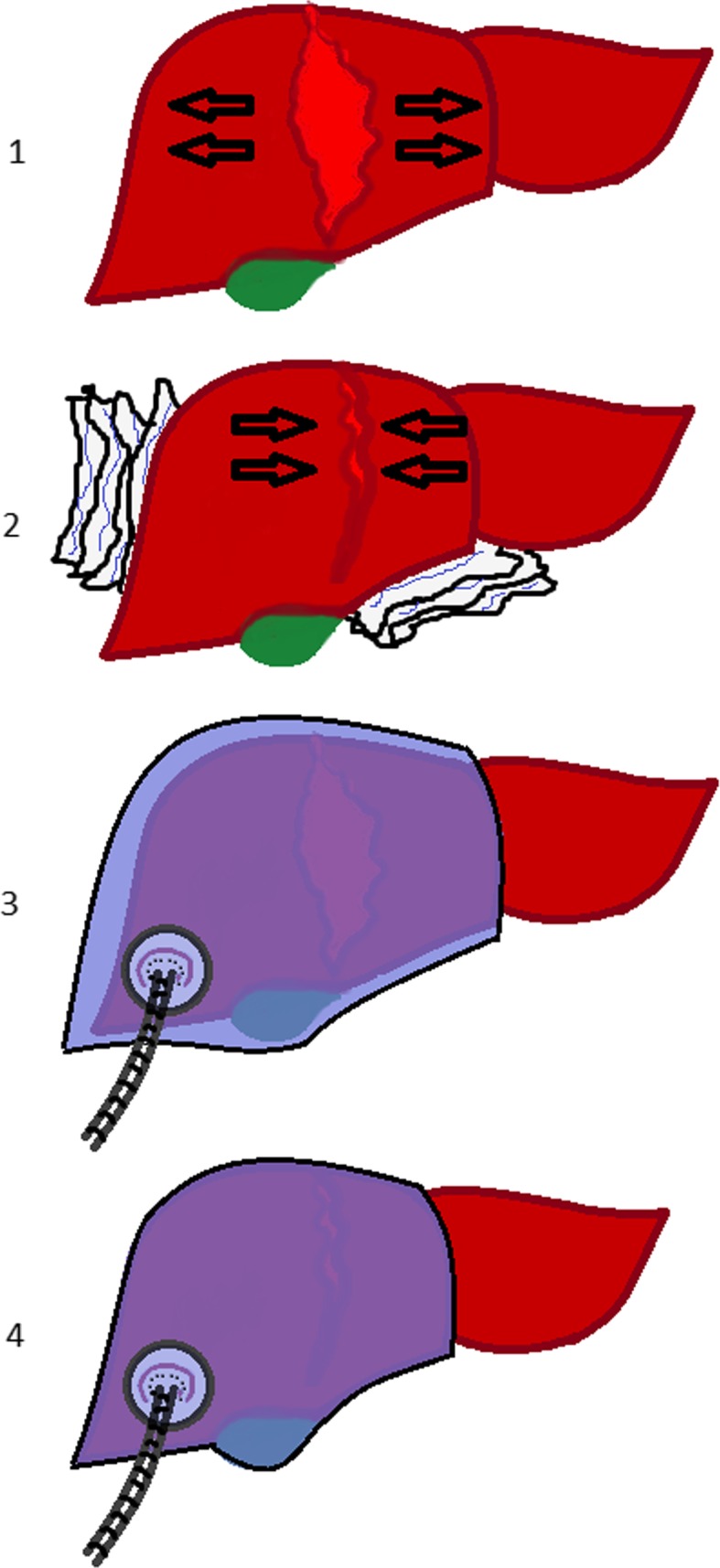
(1) Liver injury. (2) Perihepatic packing. (3) VBP placement. (4) VBP connected to vacuum, compressing the liver

**Fig. 2:**
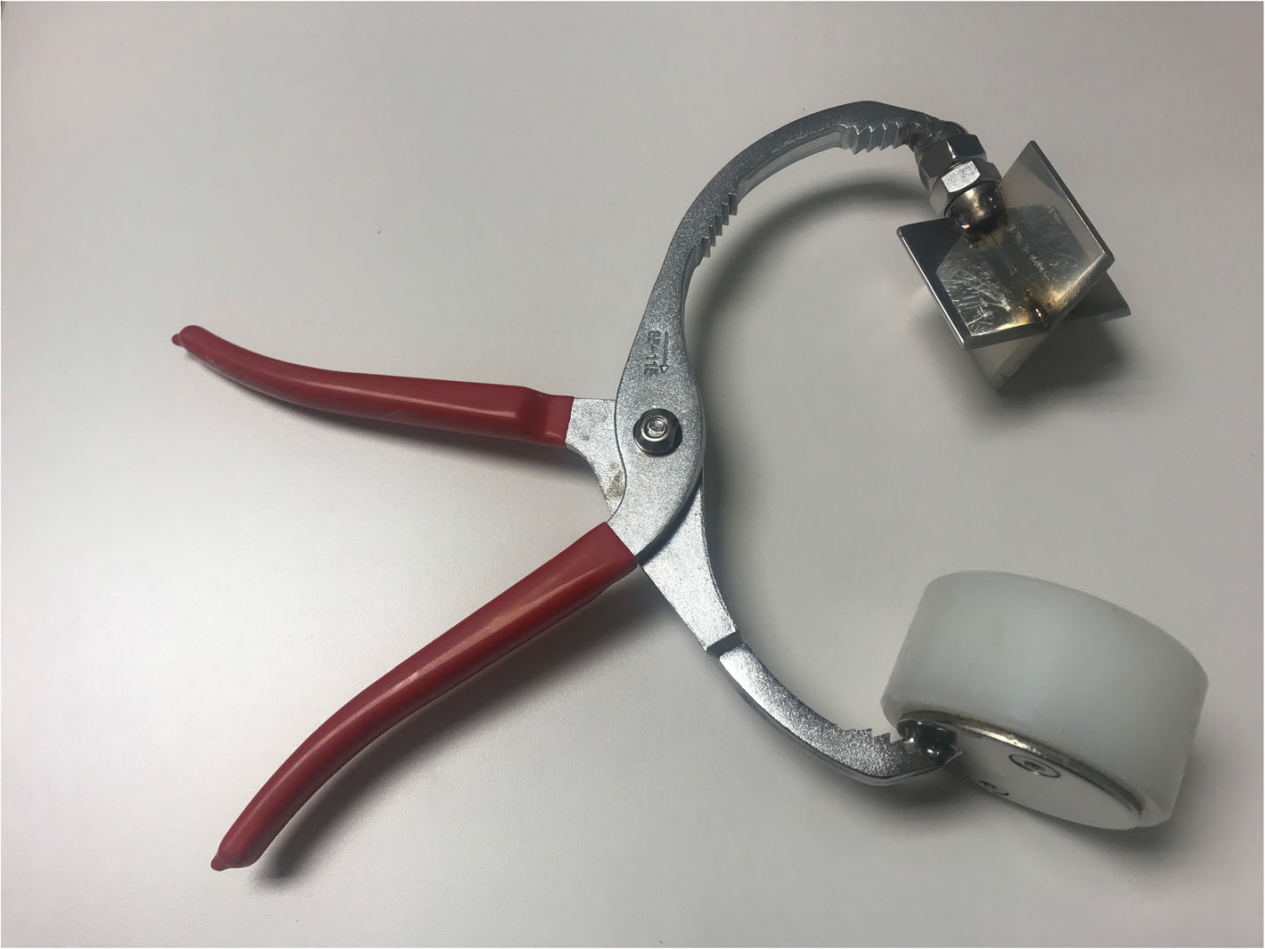
Stellate shape device used to inflict the liver injury

**Fig. 3:**
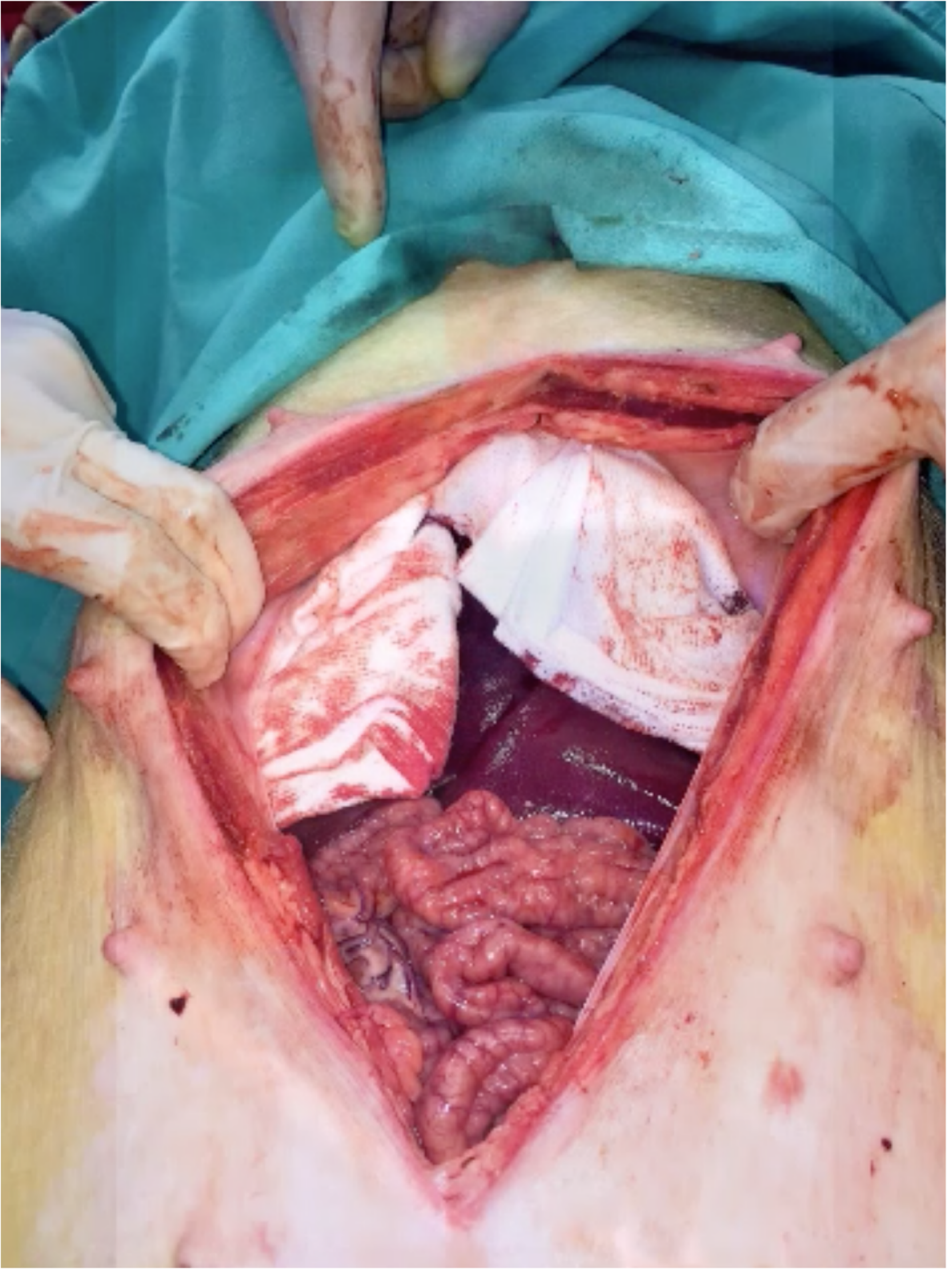
Perihepatic packing in the swine model

A xipho-pubic laparotomy was performed after sedation and analgesia of the animal by an expert veterinarian. Once the laparotomy was performed, a standard grade V liver injury, cutting the liver 2.5 cm in depth, was created with a stellate shape device as described by Holcomb et al. [13] (Figs. 1 and 2 and Additional file 2: Video 2). After that, 2 pigs were treated with PHP (Fig. 3), while the 2 remaining were treated with VBP (Additional file 3: Video 3).

**Fig. 4:**
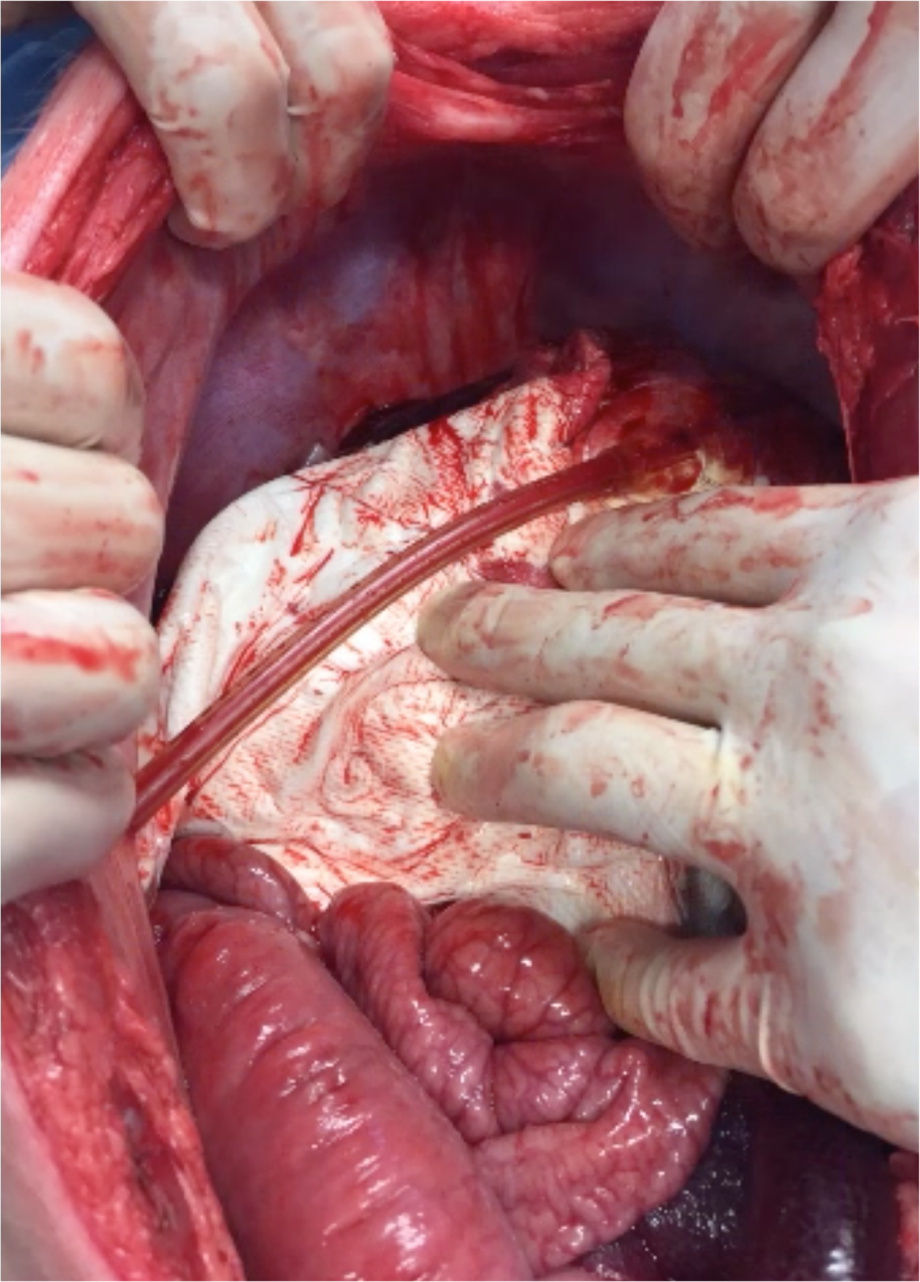
VBP placed and working in the swine model

In the PHP group, compresses were placed around the liver and the abdominal wall was subsequently closured. In the VBP group, the device was placed covering the liver. There is no need to mobilize the liver in the swine model, as the bag can easily cover a full hepatic lobe (Fig. 4). On the contrary, in humans, the liver should be partly mobilized so the bag can completely cover the injury. Once it was in place, the vacuum was established at 75 mmHg. After any of the two techniques, the laparotomy was closed using PDS loop 0.

During the surgery, aspiration and quantification of the hemorrhage was performed. In the same way, the hemodynamic stability was determined by means of an anesthesia chart as well as the need or not for vasoactive support.

### Variables

Analytical and coagulation values were determined prior to surgery as well as the reference intra-abdominal pressure (IAP). New IAP was taken after abdominal closure and daily analytical values (blood count, CRP, liver enzymes, coagulation times, fibrinogen, and lactic acid) were recorded.

After 72 h, a reintervention was performed. Both PHP and VBP were retired, and effective hemostasis and free blood in the cavity were assessed. Possible complications of the technique were described. Subsequently, the animals were euthanized in compliance with all the standards.

Continuous variables were reported as median (range), whereas categorical variables were reported as number of patients and percentage. Data were analyzed using Excel® (Microsoft 2010).

## Results

### Feasibility

The device was correctly placed without any issues during the active bleeding. Both VBP cases maintained negative pressure during 72 h with no leaks. After 72 h, the device was correctly recovered. There was no incidence of bleeding or bile leak after removal.

### Intraoperative and postoperative parameters

In the PHP group, one of the pigs did not survive the first 24 h. Both 2 pigs in the VBP group and the remaining one in the PHP group went through the full 72-h period and were successfully reoperated.

Intraoperative and postoperative parameters are exposed in Table 1. As can be seen, HR was higher in the PHP group (103 bpm) than in the VBP group (82 bpm). Temperature, IAP, and fluid requirements were similar.

**Table 1:** Vital signs and fluid requirements

	Preoperative		Postoperative		24 h		48 h		72 h	
	Packing (n:2)	VacBagPack (n:2)	Packing (n:2)	VacBagPack (n:2)	Packing (n:1)	VacBagPack (n:2)	Packing (n:1)	VacBagPack (n:2)	Packing (n:1)	VacBagPack (n:2)
HR (bpm)	75	77	94	79	82	79	73	77	103	82
Sat O2	100 %	100 %	100 %	100 %	98 %	99 %	99 %	97 %	99 %	100 %
PCO2	42	38	55	40	47	48	42	40	23	27
Tª (°C)	37.2	37.7	38	38.4	38	38.5	38	38.7	38.4	38.3
Vesical IAP	7.5	5.5	12	8.5	4	9.3	6.5	7	7	6
Fluidotherapy (ml/h)	100	100	150	100	100	100	100	100	100	100

### Blood tests

Blood test results are exposed in Table 2. Both Hb and hematocrit results were higher in the VBP group, while leukocytosis was lower. There were no substantial differences in CPR, coagulation, and liver enzymes.

**Table 2:** Blood test results

	Preoperative		Postoperative		24 h		48 h		72 h	
	Packing (n:2)	VacBagPack (n:2)	Packing (n:2)	VacBagPack (n:2)	Packing (n:1)	VacBagPack (n:2)	Packing (n:1)	VacBagPack (n:2)	Packing (n:1)	VacBagPack (n:2)
Hb (g/dl)	9.6	9.7	9.6	9.9	6.1	10	9.9	10.8	10.3	12.1
Hto (%)	30	31	30	32	19	32.15	30.4	35.4	31.7	36.7
Leucocytes (/u)	16,650	18,800	16,650	18,850	10,200	27,350	14,420	15,200	21020	17,840
CPR (mg/dL)	0.4	0.45	0.4	0.47	1.04	1.14	1.83	1.5	1.71	1.72
PT (seg)	10	13	10	9	10	11	11	11	11	10.5
GOT	43	26	250	172	270	245	167	158	110	117
GPT	46	38	46	40	62	66	64	93	67	74

## Discussion

The liver is the most injured organ following abdominal trauma and the first cause of death after suffering an abdominal injury [14].

Poor outcomes of hepatic resections in the emergency setting and the understanding of the hemorrhagic shock have led us towards the concept of damage control laparotomy with perihepatic packing. This approach, combined with advances in resuscitation and interventional radiology, has delivered a decline in overall mortality rates [15].

Perihepatic packing consists of placing compresses under pressure around the liver intended to contain the bleeding and to promote coagulation. The compresses are placed manually during the first surgical intervention. The liver is wrapped with pressure subjectively determined by the surgeon who places the compresses, based on their experience. Approximately 48–72 h after the placement of the compresses, a liver condition check is performed to check if bleeding persists. The previously placed packing is removed, and depending on the state of the liver, it can be left that way, a new surgical procedure could be needed, or in case of persisting bleeding, a new packing is placed.

Although hepatic packing is an extended maneuver, it requires experience to perform it properly and involves several potential complications. An excessive packing can cause secondary vena cava compression, which can decrease cardiac output and potentially cause death in hypotensive patients after hemorrhagic shock [2, 7]. Excessive pressure can also provoke abdominal compartment syndrome [8] with compromise of splanchnic perfusion, renal failure, heart failure, respiratory failure, and potentially death. However, a poorly placed liver packing with too low pressure may be ineffective, and thus, bleeding can continue inadvertently with the consequent hypovolemic shock and potentially death [2, 7, 9].

Even if bleeding control is achieved through this maneuver, subsequent complications such as intra-abdominal abscesses, bleeding after withdrawal, or the loss of compresses within the patient are still common.

In short, there is a need for improvements in this field to allow a simpler and safer liver packing. In particular, there is a need for devices that allow an objective way to control the pressure applied to the liver, to monitor blood debit after abdominal closure, and to prevent the loss of material within the abdominal cavity.

Inserting an organ in the bag is a relatively easy procedure that can be performed by surgeons who are not necessarily experts in abdominal trauma nor in liver surgery. The bag may thus be used as a first treatment for controlling hemorrhages in abdominal traumas. These can take place everywhere, even in places where there are no surgeons with a high expertise in abdominal traumas. Therefore, using this bag may allow for a first and quick treatment, and it provides the opportunity to refer to another center or to perform an angioembolization, which increases the probability of recovery of the patient.

Our study indicates that placing this new device is feasible. There were no leaks of the device, and no evidence of increased bleeding or bile leak due to the negative pressure was found, both of which were hypothetic risks of the new device.

Both pigs survived with the new device and showed an equivalent outcome to the one that survived in the traditional technique group. Blood tests were similar too. This suggests that VBP could be at least as effective as traditional PHP.

## Conclusions

This is a first approach and proof of concept; therefore, it has several limitations. The limited sample size in this first pilot experience and the obvious differences between pigs and humans prevent us from drawing conclusions. However, we establish a first step towards the development of this new device. A new study with different injuries in different parts of the liver and a bigger sample size still in pigs will be conducted.

Also, an industrial model of the device is currently in production.

## Supplementary information

**Additional file 1.** Video 1: Confection of a homemade prototype, the future device will not require this step as it will be a ready-to-use system.

**Additional file 2.** Video 2: Stellate shape device inflicting the liver injury

**Additional file 3.** Video 3: VBP placed and activated in order to stop the hepatic bleeding

## Data Availability

Excel documents’ photographies and videos are available.

## Abbreviations

CRP: C-reactive protein
IAP: Intrabdominal pressure
PHP: Perihepatic packing
VBP: VacBagPack device
